# Using a HIV prevention cascade for identifying missed opportunities in PrEP delivery in Kenya: results from a programmatic surveillance study

**DOI:** 10.1002/jia2.25537

**Published:** 2020-06-30

**Authors:** Daniel Were, Abednego Musau, Jane Mutegi, Patricia Ongwen, Griffins Manguro, Mercy Kamau, Tom Marwa, Hellen Gwaro, Irene Mukui, Marya Plotkin, Jason Reed

**Affiliations:** ^1^ Jhpiego Nairobi Kenya; ^2^ International Center for Reproductive Health Mombasa Kenya; ^3^ National AIDS and STI Control Program Nairobi Kenya; ^4^ Jhpiego Baltimore MD USA

**Keywords:** PrEP, cascades, HIV prevention, key and vulnerable populations, missed opportunities, sub‐Saharan Africa

## Abstract

**Introduction:**

HIV prevention cascades have been systematically evaluated in high‐income countries, but steps in the pre‐exposure prophylaxis (PrEP) service delivery cascade have not been systematically quantified in sub‐Saharan Africa. We analysed missed opportunities in the PrEP cascade in a large‐scale project serving female sex workers (FSW), men who have sex with men (MSM) and adolescent girls and young women (AGYW) in Kenya.

**Methods:**

Programmatic surveillance was conducted using routine programme data from 89 project‐supported sites from February 2017 to December 2019, and complemented by qualitative data. Healthcare providers used nationally approved tools to document service statistics. The analyses examined proportions of people moving onto the next step in the PrEP continuum, and identified missed opportunities. Missed opportunities were defined as implementation gaps exemplified by the proportion of individuals who could have potentially accessed each step of the PrEP cascade and did not. We also assessed trends in the cascade indicators at monthly intervals. Qualitative data were collected through 28 focus group discussions with 241 FSW, MSM, AGYW and healthcare providers, and analysed thematically to identify reasons underpinning the missed opportunities.

**Results:**

During the study period, 299,798 individuals tested HIV negative (211,927 FSW, 47,533 MSM and 40,338 AGYW). Missed opportunities in screening for PrEP eligibility was 58% for FSW, 45% for MSM and 78% for AGYW. Of those screened, 28% FSW, 25% MSM and 65% AGYW were ineligible. Missed opportunities for PrEP initiation were lower among AGYW (8%) compared to FSW (72%) and MSM (75%). Continuation rates were low across all populations at Month‐1 (ranging from 29% to 32%) and Month‐3 (6% to 8%). Improvements in average annual Month‐1 (from 26% to 41%) and Month‐3 (from 4% to 15%) continuation rates were observed between 2017 and 2019. While initiation rates were better among younger FSW, MSM and AGYW (<30 years), the reverse was true for continuation.

**Conclusions:**

The application of a PrEP cascade framework facilitated this large‐scale oral PrEP programme to conduct granular programmatic analysis, detecting “leaks” in the cascade. These informed programme adjustments to mitigate identified gaps resulting in improvement of selected programmatic outcomes. PrEP programmes are encouraged to introduce the cascade analysis framework into new and existing programming to optimize HIV prevention outcomes.

## 1 INTRODUCTION

Despite the strides made in fighting the HIV epidemic, globally an estimated 1.7 million people still acquire HIV infection annually [1]. Kenya has the fifth largest HIV epidemic in the world. By 2018, an estimated 1.3 million adults were living with HIV, with an estimated 36,000 new adult HIV infections annually [2]. In the last decade, HIV incidence in the general population in Kenya has stabilized or fallen, but key populations, including female sex workers (FSW), men who have sex with men (MSM) and persons who inject drugs (PWID) continue to experience a high burden of HIV and influence the HIV transmission dynamics. FSW, MSM and PWID accounted for 15% of new HIV infections in East and Southern Africa (ESA) in 2018 [1]. Similarly, a third of all new HIV infections in Kenya are attributed to FSW, MSM and PWID [3].

Globally, adolescent girls and young women (AGYW) aged 15 to 24 years are disproportionately affected by the HIV epidemic and accounted for 60% of the estimated 510,000 new HIV infections within that age group in 2018 [1, 4]. AGYW experience a higher vulnerability compared to their male counterparts due to biological and socio‐cultural factors, gender and power dynamics and economic disadvantage [4]. In sub‐Saharan Africa (SSA), AGYW represent 10% of the population, but contribute one in every five new HIV infections [5]. Furthermore, AGYW accounted for 26% of all new HIV infections in Eastern and Southern Africa in 2018 [1]. AGYW in Kenya were estimated to account for 28% of new HIV infections among individuals 15 years and older in 2017 [6]. Kenya’s epidemic is geographically heterogeneous hence majority of these new infections were concentrated in a few counties with high HIV incidence in the general population [6].

Oral pre‐exposure prophylaxis (PrEP) is being rolled out in many SSA countries [7] following WHO’s 2015 recommendation [8]. Kenya issued guidelines in 2016 promoting oral PrEP as part of a comprehensive package of HIV prevention services, including condoms, voluntary medical male circumcision (VMMC), treatment as prevention, stigma and violence prevention, post‐exposure prophylaxis, among others [9], and subsequently launched national scale‐up in 2017 [10]. To reach FSW and MSM, Kenya’s national HIV programme provides services mainly through community‐led drop in centres (stand‐alone clinics that primarily provide HIV prevention, care and treatment services to FSW and MSM) and peer outreach [11, 12, 13]. Additionally, AGYW are served through public and private health facilities that integrate sexual and reproductive health (SRH) services. Since its adoption, PrEP is delivered for FSW, MSM and AGYW through these integrated platforms [10, 14].

Cascades have been used extensively to quantify steps [15, 16] and identify gaps in service delivery, including for HIV care and treatment services [17, 18] as well as tuberculosis [19]. HIV prevention cascades are now being proposed to strengthen HIV prevention programmes, and a number of cascades have been developed [20, 21, 22, 23]. Available cascades have been developed using routinely collected programme data [22], population‐based survey data [24], or a blend of the two data sources [11].

In a PrEP cascade, quantifiable benchmarks are measured as clients move through the process of receiving services, from initial HIV testing, risk and clinical eligibility screening, to PrEP initiation and continuation [25]. Measurement in a PrEP cascade and interpretation of gaps is complicated by challenges related to the temporality and typology of individuals’ risk behaviours, that is despite being in a high HIV risk category, not all people taking PrEP are continuously at substantial risk and so may or may not fully progress through the cascade, even if using PrEP effectively [26]. Furthermore, individuals may switch to alternative HIV prevention interventions hence a holistic cascade that incorporates complementary prevention interventions is desirable [24]. Although PrEP cascades have been operationalized in developed countries [27, 28, 29], it is only recently that PrEP cascades are increasingly emerging in SSA [24, 30, 31]. Furthermore, the majority are derived using research data. To the best of our knowledge, there are no published examples of PrEP cascades using routine programmatic data from the region.

This paper presents an analysis of programmatic surveillance data from a large PrEP scale‐up project for FSW, MSM and AGYW in Kenya. The objectives of the study were as follows: to describe the programmatic application of an oral PrEP cascade; to quantify progression across each step of the cascade for FSW, MSM and AGYW beneficiaries of the project, looking at differences between populations; and, to identify missed opportunities and programme implications from trends in each step.

## 2 METHODS

**Figure 1:**
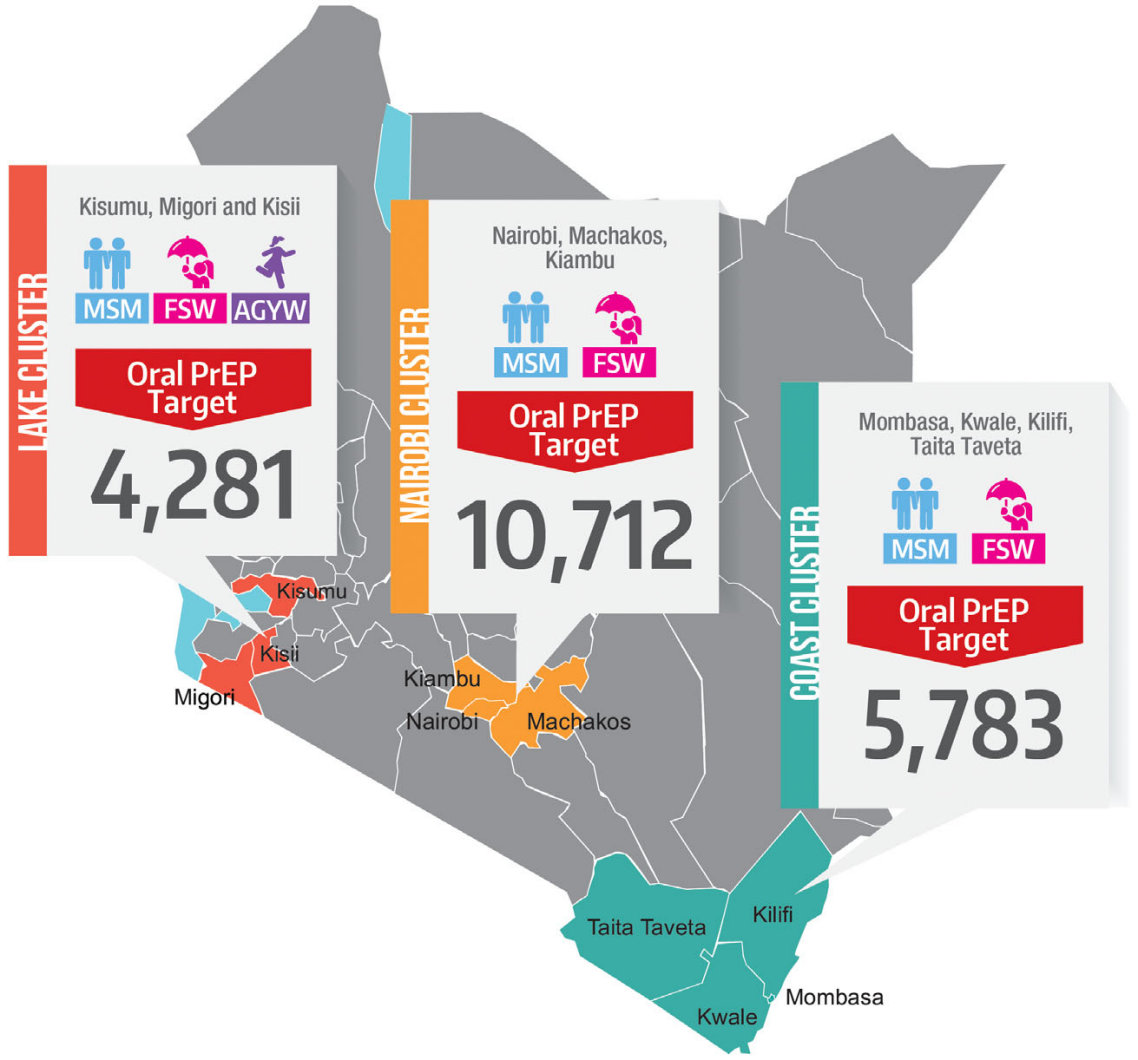
Geographical coverage and oral PrEP targets of the Jilinde Project in Kenya. Source: Jilinde Project. PrEP, pre‐exposure prophylaxis.

This analysis was conducted using data collected from February 2017 to December 2019 in three geographical clusters (Lake, Nairobi and Coast) in Kenya (Figure 1), in the context of a large‐scale routine service delivery project providing PrEP to FSW, MSM and AGYW. The study design was a programmatic surveillance approach [32] using routine service delivery and qualitative data.

### 2.1 Project description

Jilinde (Bridge to Scale) is a five‐year project, which began in July 2016, implemented in collaboration with the Ministry of Health (MOH) of Kenya. Jilinde is scaling up oral PrEP through integration into routine health services in drop‐in centres (DICEs), public and private health facilities. The project, implemented in 10 out of the 47 counties in Kenya, aimed to enrol 15,500 FSW, 3300 MSM and 2000 AGYW on PrEP (Figure 1). Project targets were set with the goal of enrolling 15% of all FSW and 33% of all MSM in nine out of the ten counties of Kenya on PrEP based on national size estimates [33], whereas AGYW targets were exploratory to establish interest in PrEP uptake among AGYW, and only in one county (Migori).

**Figure 2:**
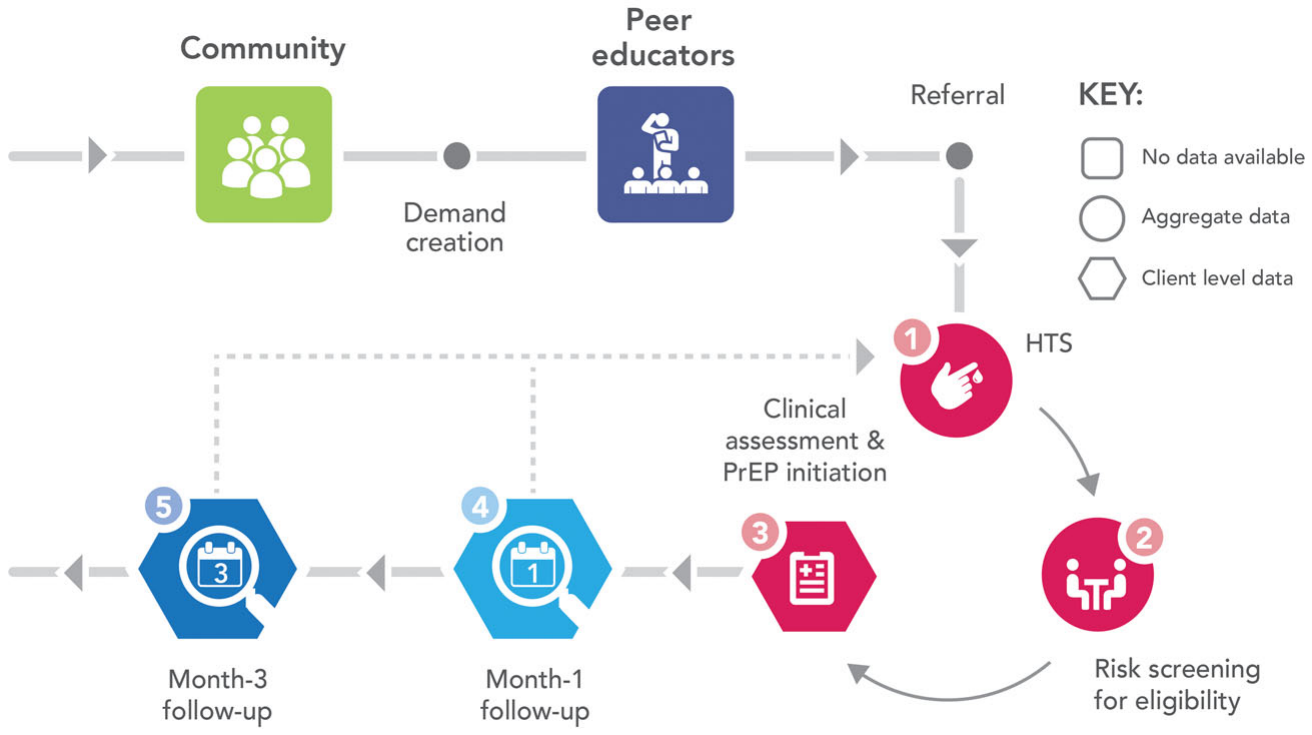
PrEP service delivery pathway in the Jilinde project – PrEP cascade incorporates steps 1 to 5. Source: Jilinde Project. PrEP, pre‐exposure prophylaxis.

Individuals enter the PrEP pathway illustrated in Figure 2 through community mobilization mainly conducted by peer educators trained by the project. Individuals who express interest in PrEP are referred to the different facilities providing PrEP, where they undergo HIV testing services (HTS) following the national guidelines [34]. Upon confirmation of negative HIV status, clients undergo a behavioural risk screening. Those who screen positive for substantial behavioural risk, or who request PrEP, are referred to an onsite clinician who conducts a clinical assessment and provides PrEP to clients who are eligible and opt‐in [10]. Typically, HTS, eligibility screening and clinical assessment occur on the same day. Clients enrolled on PrEP are scheduled for a follow‐up visit at the same facility one month following initiation and monthly thereafter, and are provided with the option to access adherence support interventions including: short message service (SMS) reminders; phone call reminders; PrEP support groups and adherence buddies.

### 2.2 Data sources and analysis

We used two main data sources; routine programmatic data to operationalize the PrEP cascade, and qualitative data to identify underlying reasons for the implementation gaps.

#### 2.2.1 Routine programmatic data

Data were collected from 89 sites (34 DICEs, 42 public health facilities and 13 private health facilities) in the 10 counties where the Jilinde project operates. The data were collected in the context of routine service delivery, with standardized tools approved by the MOH. These include the national HTS registers (for first three steps of the cascade: tested for HIV, tested HIV negative and screened for PrEP eligibility) and client encounter forms (for last three steps of the cascade: initiation on PrEP, attendance at Month‐1 and Month‐3 follow‐up) as summarized in Figure 2. Healthcare providers (HTS providers, clinical officers and nurses) provided HTS and PrEP and recorded information in the HTS registers, which are aggregated into a site‐level monthly report. PrEP initiation and follow‐up data from the client encounter forms was monitored longitudinally at the client level. Monthly data verification and quarterly data quality audits were conducted to ensure data quality.

The analyses for the cascade examines simple proportions of people moving onto the next step in the PrEP continuum. The starting point is the cumulative number of individuals who underwent HIV testing. The first three steps of the cascade are computed using aggregate data generated from national HIV testing tools, which summarize the counts of individuals who progressed through the steps. The subsequent steps (initiated PrEP, Month‐1 and Month‐3 follow‐up) are computed using client‐level data that accounts for unique individuals. A PrEP refill within 37 and 97 days of scheduled return at successful follow‐up visit at one and three‐month post‐start served as a proxy for continued PrEP use at Month‐1 and Month‐3 respectively. The proportions for each step of the cascade are computed using the number of individuals reaching that step as the numerator, and the number who reached the previous step in the cascade as a denominator.

The cascade indicators are defined as follows: Tested HIV negative is the aggregate number of individuals whose HIV test results were negative as a proportion of the cumulative number of individuals who underwent an HIV test. Screened is the proportion of individuals who underwent behavioural risk screening among individuals whose HIV test results were negative, whereas eligible is the proportion of individuals who met eligibility criteria for PrEP among individuals screened. Initiated PrEP is the number of individuals who were prescribed and dispensed a 30 days’ supply of PrEP as a proportion of the cumulative number of individuals who were eligible for PrEP. Month‐1 follow‐up is the number of individuals who had returned for a PrEP refill as a proportion of the number of individuals who had completed 37 days’ post‐PrEP initiation. Month‐3 follow‐up is the number of individuals who had returned for a PrEP refill as a proportion of the number of individuals who had completed 97 days’ post‐PrEP initiation.

“Missed opportunities,” were proportions of individuals who could have potentially accessed each step in the PrEP continuum who did not. Missed opportunities include individuals who tested HIV negative and could have been screened, but were not; those who were eligible for PrEP, but did not initiate and those who initiated PrEP, but did not return on time for their Month‐1 and Month‐3 follow‐up visit. The identification of missed opportunities and institution of programme adjustments was implemented continuously throughout the surveillance period. To detect changes in the indicators at various points during the surveillance period, we compared the proportions of the cascade indicators across different months from February 2017 to December 2019.

#### 2.2.2 Qualitative data

Data were collected between October 2017 and May 2019 through 28 focus group discussions (FGDs). Participants included 44 FSW, 59 MSM, 91 AGYW and 47 healthcare providers, who were purposively selected from project‐supported sites. FGDs were moderated by trained sex‐matched qualitative researchers in English, Kiswahili or local language (Dholuo) and conducted in privacy. FGDs were audio‐recorded and handwritten notes taken. The handwritten notes were typed and audio files transcribed verbatim. The transcripts for FGDs conducted in Kiswahili and Dholuo were translated to English. Thematic analysis was conducted using Nvivo 11. Emergent themes are summarized in Table 2.

### 2.3 Ethical considerations

Ethical oversight was provided by the Kenya Medical Research Institute (KEMRI) institutional review board (IRB) and a non‐research determination was obtained from the Johns Hopkins Bloomberg School of Public Health IRB.

## 3 RESULTS

### 3.1 Overall PrEP cascade

**Figure 3:**
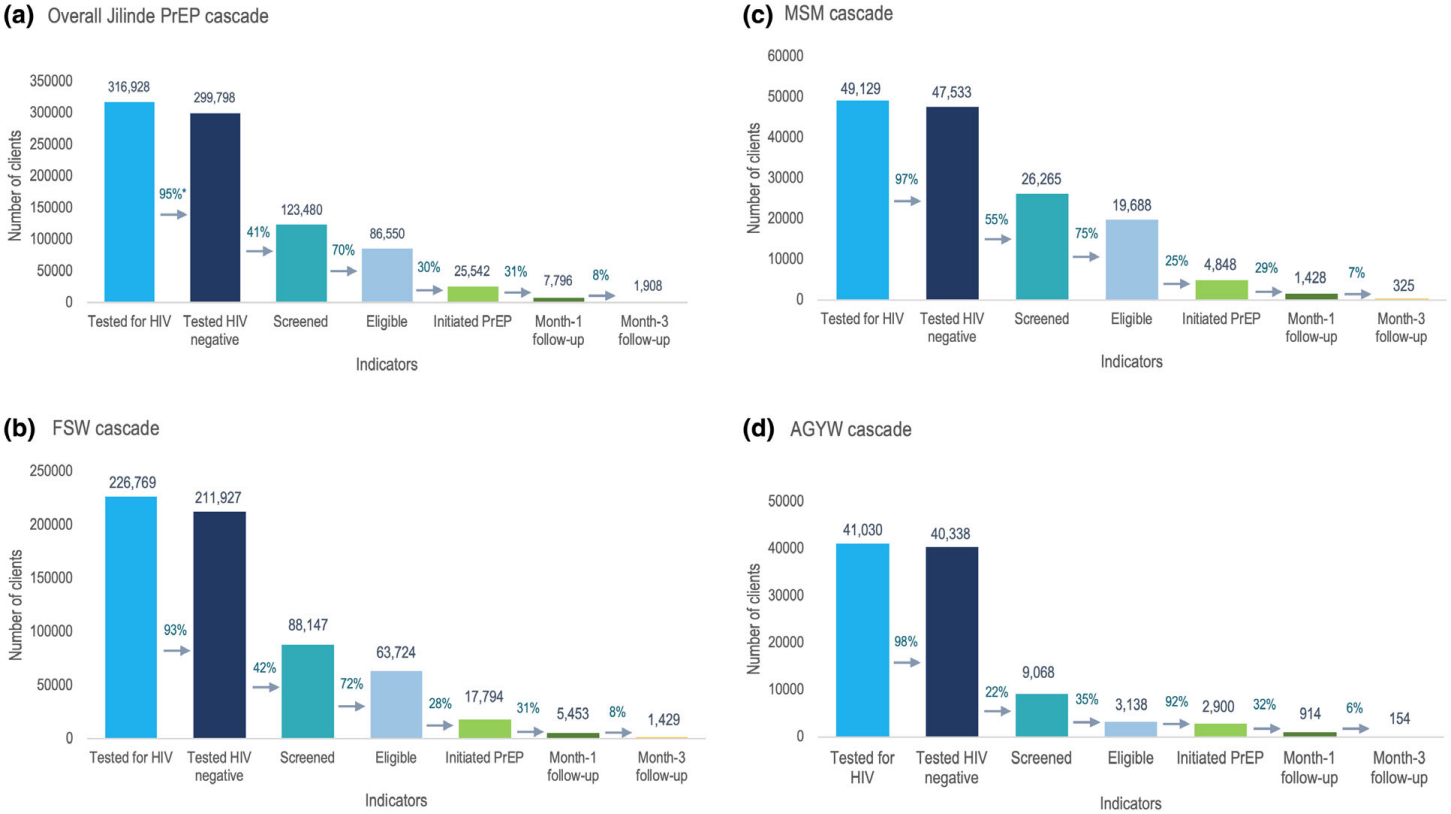
Overall Jilinde project (A) and population‐specific (B, FSW; C, MSM; D, AGYW) PrEP cascades from February 2017 to December 2019. *% denotes proportion of clients proceeding to the next step in the cascade. PrEP, pre‐exposure prophylaxis.

Between February 2017 and December 2019, 316,928 HIV tests were performed, of which 299,798 (95%) were HIV negative, though an unquantified level of repeat testing occurred. From these testing activities, 123,480 HIV‐negative individuals were screened for PrEP, representing at least 41%, the exact estimate dependent upon the extent of repeat testing, and 86,550 (70%) were eligible for PrEP (Figure 3A). Among PrEP‐eligible individuals, 25,542 (30%) consisting 17,794 (70%) FSW, 4,848 (19%) MSM and 2,900 (11%) AGYW, were initiated on PrEP, exceeding the project targets for FSW and MSM in Figure 1. Of these clients, 61% of FSW and 42% of MSM were above 24 years, whereas 55% of AGYW were between 20 and 24 years. Majority (81%) of clients initiated PrEP through DICEs, whereas 14% and 5% were initiated through public and private facilities respectively. Among initiates 7,796 (31%) returned for their Month‐1 follow‐up, and 1,908 (8%) for their Month‐3 follow‐up visits (Figure 3A). Cascades for the three population types are summarized in Figure 3B,C,D.

### 3.2 Missed opportunities in the FSW, MSM and AGYW PrEP cascades

**Figure 4:**
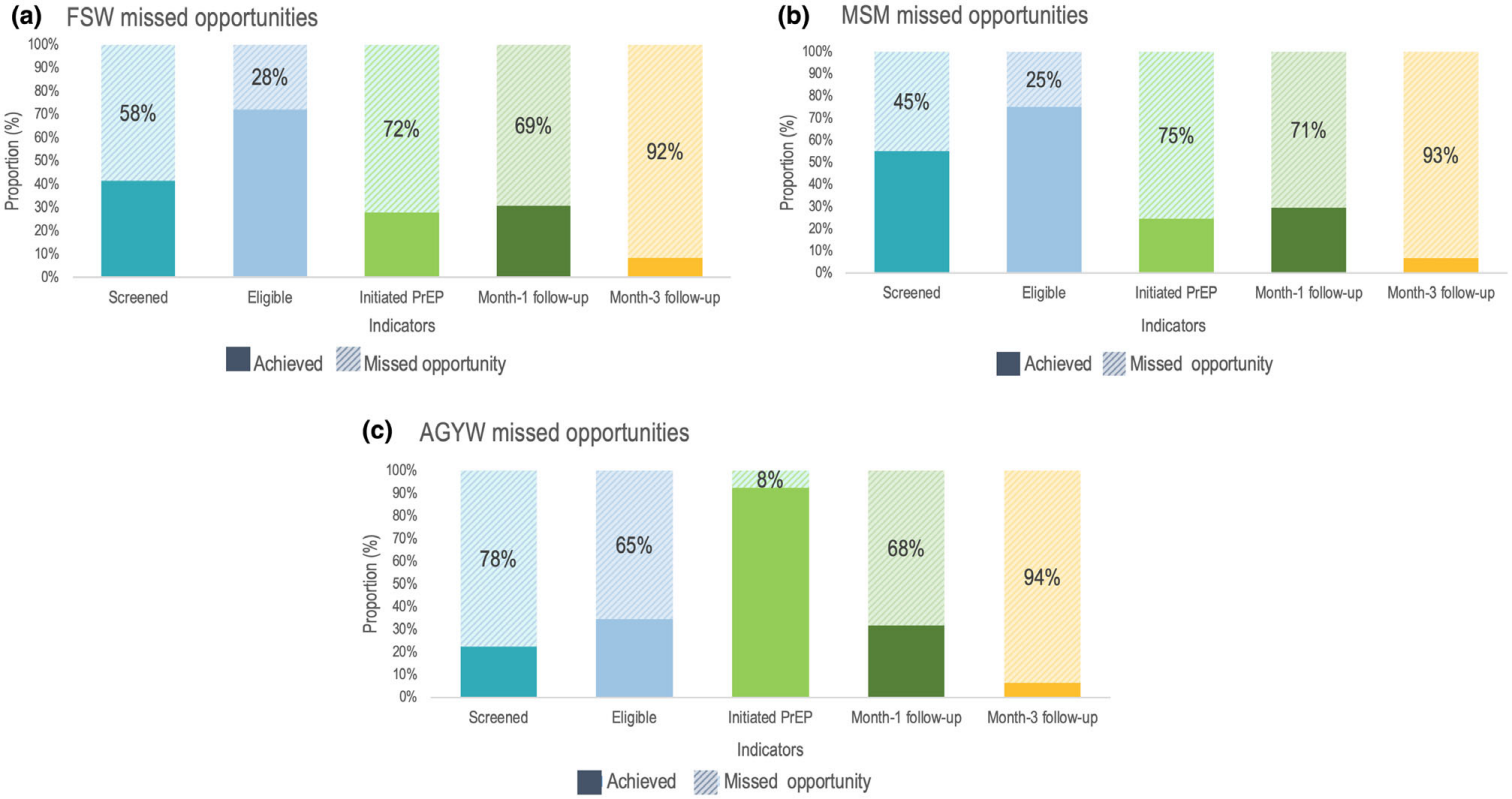
Missed opportunities in the FSW (A), MSM (B) and AGYW (C) PrEP cascades, July 2017 to December 2019. AGYW, adolescent girls and young women; FSW, female sex workers; MSM, men who have sex with men; PrEP, pre‐exposure prophylaxis.

AGYW had higher missed opportunities for screening (78%) compared to FSW and MSM (Figure 4). There was a greater proportion of FSW who were not screened compared to MSM, 58% versus 45%. Among those screened, a substantially higher proportion of AGYW (35%) were ineligible for PrEP compared to 28% of FSW and 25% of MSM. Missed opportunities for PrEP initiation were higher for FSW (72%) and MSM (75%) compared to AGYW (8%). Majority of clients did not persist on PrEP use at Month‐1 (69% of FSW, 71% of MSM and 68% of AGYW) and Month‐3 (92% of FSW, 93% of MSM and 94% of AGYW) follow‐ups.

### 3.3 Cascades for FSW, MSM and AGYW disaggregated by age

When disaggregated by age, there were mixed variations in the proportion of individuals screened for PrEP and those found eligible for PrEP across the age groups for the three population types as summarized in Table 1. The proportion of individuals initiated on PrEP was highest among FSW and MSM below 20 years and lowest among individuals older than 30 years. Month‐1 and Month‐3 follow‐up rates were slightly higher among older FSW and minimal variation was observed for AGYW.

**Table 1:** Cascade indicators for FSW, MSM and AGYW disaggregated by age groups (years)

	FSW				MSM				AGYW	
	15 to 19	20 to 24	25 to 30	>30	15 to 19	20 to 24	25 to 30	>30	15 to 19	20 to 24
Tested HIV Negative, %	99	99	98	86	98	97	98	92	99	98
Screened, %	37	39	44	42	58	56	58	51	22	23
Eligible, %	74	75	73	70	75	76	74	75	36	34
Initiation, %	37	35	29	21	36	28	22	18	95	91
Month‐1 follow‐up, %	25	29	32	33	31	28	30	33	31	32
Month‐3 follow‐up, %	6	7	8	10	8	6	7	8	5	5

AGYW, adolescent girls and young women; FSW, female sex workers; MSM, men who have sex with men; PrEP, pre‐exposure prophylaxis.

### 3.4 Trends in the PrEP cascade

**Figure 5:**
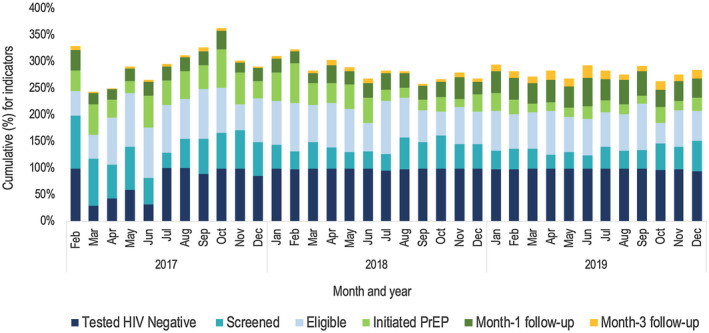
Trends in the Jilinde PrEP cascade indicators from February 2017 to December 2019.

There were month‐by‐month variations across all the cascade indicators as summarized in Figure 5. The proportion of individuals reported to have tested HIV negative was lowest in March to June 2017. Thereafter, the annual average proportion of those testing HIV negative was 95% in 2017 and remained at 98% in 2018 and 2019. However, an annual decline in the proportion of individuals who were screened for PrEP eligibility (from 67% in 2017, 44% in 2018 to 37% in 2019) was observed. The proportion of individuals eligible for PrEP declined from an annual average of 77% in 2017, to 73% in 2018 and 68% in 2019. Additionally, those initiated on PrEP declined from 47% in 2017, to 36% in 2018 and 22% in 2019. The annual average Month‐1 follow‐up rate increased from 26% in 2017, to 27% in 2018 and 41% in 2019. Month‐3 follow‐up rates increased from 4% in 2017, to 6% in 2018 and 15% in 2019.

### 3.5 Contextual findings on missed opportunities and programmatic adjustments

In addition to the population‐specific missed opportunities presented in Figure 4, contextual factors underpinning the missed opportunities derived through qualitative research, and specific programmatic adjustments made by Jilinde or by the MOH are presented in Table 2.

**Table 2:** Missed opportunities and contextual findings for Jilinde project

Missed opportunity in the PrEP cascade	Key themes from qualitative research	Sample quotes from FGDs	Continuous programmatic adjustments by Jilinde project
Screening for PrEP	Routine risk screening is low priority for HIV testing services (HTS) providers	*“…… we have a lot of work when a patient comes all you want to do is treat the patient. Focus on whatever brought the patient and you do not want to explore”* (*41‐year‐old female clinician from Migori*)	Advocacy to county health management teams through technical working groups and review meetings to enhance accountability for PrEP indicators
	PrEP screening conducted, but not recorded due to frequent changes in the documentation tools	“*…. you screen yet you fail to indicate that you have screened. It is a challenge……… if you don’t document someone can easily say you did not screen.”* (*27‐year‐old male HTS Provider from Migori)*	Jilinde advocated and the MOH revised the national HIV Testing Services Register (MOH 362) to capture eligibility screening
Eligibility for PrEP	Poor rapport between AGYW and providers inhibits disclosure of risk behaviours	*“… if we create a positive environment during the time we are with the client, then the client will not feel uncomfortable but if our starting point was not structured well, then this is where we get a client who will never be ready to disclose or to be open*” (*27‐year‐old male HTS Provider from Migori)*	Trained healthcare providers on delivery of youth‐friendly services to make them more sensitive to AGYW
	Peer mobilization and referral of low risk individuals coupled with inadequate client education on PrEP	“*As for the youth peer providers (YPPs) try to link them (AGYW)…… even if they have the knowledge it is not that powerful to help us in terms of getting the right clients for PrEP.”* (*36 year old male HTS provider from Migori*)	Trained mobilizers on communication skills, introduced “stages of change” tool
Initiation of PrEP	Myths and misconceptions about PrEP and low risk perception among FSW, MSM and AGYW	*“In MSM circles they say those who take PrEP are people who love sex a lot, they are addicted to it” (23‐year‐old MSM from Nairobi)*	Implementation of user‐centered demand creation strategies utilizing social networks; recruitment and training of trusted peer educators to mobilize and refer their peers for PrEP
	Co‐location of both PrEP and HIV services in comprehensive care centers (HIV clinics) resulted in PrEP clients feeling stigmatized as HIV positive	*“In our facility we've come to realize that most patients who are on PrEP don't like being mixed with HIV positive, so they don't like accessing PrEP at the comprehensive care centre (CCC), at times they prefer they access from the outpatient” (25‐year‐old female nurse from Machakos)*	Integrated PrEP with SRH services for AGYW; piloted DICE for AGYW; diversified service provision through community safe spaces, hotspots, outpatient departments, maternal child health and family planning clinics
	Stigma‐related discouragement from peers, family and friends for eligible users	*“…parents discourage people not to take PrEP; they can say that this drug will increase immorality in the community” (21‐year‐old AGYW from Migori)*	Mass media, social media and promotional events to create a supportive environment for PrEP uptake
	Providers reluctance to prescribe PrEP associated with reluctance to increase provider workload; provider belief that client will not adhere to PrEP	*“… in our case is work load, we have one clinician who is supposed to do the outpatient, clear the queue outside there, at the same time is the person who is supposed to operate in the CCC. At times he gets tired and tells them ‐ you come tomorrow for PrEP” (29‐year‐old male clinician from Machakos)*	Testimonies from champion providers and satisfied users to champion for PrEP delivery during facility review meetings
	Insensitive referral and access pathways in public and private health facilities	*“The testing place is different from the place I was asked questions and the place for collecting the medicine is also different. We took long because we were walking from one place to another” (22‐year‐old AGYW from Migori)*	Instituted continuous quality improvement to identify and address gaps in the PrEP delivery pathway; site level supervision, mentorship and coaching
Continuation	Clients experiencing side effects, ambivalence and low intrinsic motivation to use prevention interventions	*“I was getting tired. I felt that the body could not make it. I wanted to go to work but I was unable. I just wanted to sleep and that made me angry and I stopped using them” (35‐year‐old FSW from Mombasa)*	Implemented persistence support interventions: pre‐initiation counselling and readiness assessment, follow‐up calls, SMS reminders, peer educators tracked clients lost‐to‐follow up and assisted clients to identify adherence buddies
	Myths and misconceptions about PrEP, stigma and negative peer influence	*“What made me to stop taking PrEP was my two friends who said that I was HIV positive…” (22‐year‐old, FSW from Kisii)*	Developed and disseminated frequently asked questions (FAQs) about PrEP brochures, established PrEP support groups and sharing testimonials from satisfied PrEP users
	Operational and access barriers (Long waiting time, arduous referral pathways)	*“It takes a lot of time because you find many people in a line. Sometimes people are busy there’s no one to attend to you; you move from one place to another...” (22‐year‐old AGYW from Migori)*	System‐level adjustments to improve the efficiency of PrEP services (PrEP refill days, community‐based refills, integrated services, multi‐month scripting)
	Provider perceptions (negative attitudes, stereotypes and discrimination)	*“Experienced nurses and clinicians at the facility I work are elderly and when they hear about giving someone the drugs, they will tell you ''Change young man. Go and change.'' They can't give you the drug.” (29‐year‐old male clinician from Nairobi)*	Trained healthcare providers on gender, sexual diversity and value clarification
	Unappealing branding (Similar to ARVs for treatment), and packaging (rattling pill bottle)	*“…the way PrEP is; its branding is like ARVs. Someone can think I am positive because I have drugs …. So it is a problem and it is interfering with adherence. You take and stop because people are talking a lot, insults,” (22‐year‐old MSM PrEP client from Nairobi)*	Piloted use of alternative and client‐friendly pill carriers developed through human‐centred design

AGYW, adolescent girls and young women; DICE, drop‐in centre; FGDs, focus group discussions; FSW, female sex workers; HTS, HIV testing services; MOH, Ministry of Health; MSM, men who have sex with men; PrEP, pre‐exposure prophylaxis; SMS, short message service; SRH, sexual and reproductive health.

## 4 DISCUSSION

In this paper, we describe how the Jilinde project used PrEP cascade analysis to describe coverage and identify missed opportunities for oral PrEP among FSW, MSM and AGYW clients in routine settings where PrEP is integrated with other HIV prevention services. The study affirms the feasibility of constructing cascades using programmatic data complemented by qualitative enquiry to understand where and why implementation gaps exist, which can guide programming changes. From this study, the three main missed opportunities identified were screening for PrEP eligibility, PrEP initiation and follow‐up. These varied across the different steps in the cascades, by age group, population type and during different time periods.

PrEP is nascent in SSA and many countries are in the formative stages of designing effective implementation approaches [7]. In the absence of implementation evidence on PrEP delivery at scale in SSA, HIV prevention programmes integrating PrEP can use programmatic and implementation research data to adapt and optimize implementation [35]. Countries can employ the cascade framework to facilitate critical programmatic analysis and institute necessary changes. Furthermore, complementary data sources have potential to generate in‐depth explanations of the gaps, and suggest interventions needed to optimize implementation. The cascade approach employed in this analysis is comparable to studies that have developed cascades for various HIV prevention interventions such as PrEP, condoms and VMMC [21, 23, 31].

This study has established gaps in screening HIV‐negative individuals for PrEP eligibility, similar to other programmes delivering PrEP [35, 36]. Plausible explanations for the screening gaps for all populations, and especially AGYW, include prioritization of HIV case finding and huge workload occasioned by shortage of healthcare workers, which is consistent with previous studies in Kenya and elsewhere [36, 37, 38, 39, 40, 41]. Furthermore, the rapid scale up of PrEP in Kenya may have contributed to increased volume of HIV testing [14] reducing the propensity for HTS providers to offer eligibility screening to every HIV‐negative client. Additionally, poor documentation attributed to shifting MOH guidance on behavioural risk screening tools and procedures may have contributed to the screening gaps identified.

PrEP initiation among eligible individuals was consistently low except for AGYW. The latter contrasts previous studies which have reported low PrEP uptake among AGYW [42, 43, 44]. The high PrEP uptake among AGYW could be attributed to targeted mobilization of potentially eligible AGYW through peer educators, integration of PrEP with sexual and reproductive services and implementation of community delivery models. This is congruent with existing literature which recommends that PrEP should be integrated into comprehensive programmes and delivery simplified to make it attractive to AGYW [44, 45]. The consistently low PrEP uptake among FSW and MSM over the three‐year period is consistent with studies globally, which report a high interest in PrEP, but low uptake [46, 47, 48]. Similar to the findings from the qualitative inquiry, studies have elucidated client‐level and health system barriers, and perceived negative experiences when accessing or using PrEP as disincentives for PrEP uptake in numerous settings [49, 50, 51, 52]. Furthermore, FSW and MSM were likely to be accessing alternative HIV prevention interventions, which they might have preferred over PrEP. This is corroborated by a study in Zimbabwe which elucidated that preference for condoms impeded PrEP uptake [53], whereas FSW in Kenya have reported high rates of condom use [11]. These findings suggest that intensive demand creation is essential to normalize PrEP and improve uptake as witnessed in the United States [47].

Overall continuation rates in the Jilinde cascade were low, compared to research studies and demonstration projects [47, 50, 52, 54]. There is a paucity of evidence on continuation rates for FSW, MSM and AGYW from HIV prevention programmes that have scaled‐up PrEP in SSA, and our findings may provide a more accurate illustration of continuation rates within a real‐world scale‐up context. Given that continuation is a proxy indicator for effective PrEP use, these continuation rates could be considered suboptimal given the risk profile of FSW, MSM and AGYW from geographies with high HIV incidence in the general population. It is plausible that missed opportunities for continuation may be over‐estimated because the cascade did not document individuals who self‐discontinued PrEP because their risk profile changed, or substituted PrEP with alternative prevention methods, given that PrEP was delivered in the context of a broader prevention programme. Findings from the qualitative research revealed that lack of motivation and commitment, stigma, product‐related and health system challenges were explanations for the low PrEP continuation. These findings are consistent with studies which have documented similar barriers to PrEP continuation [44, 49, 52]. The programmatic interventions to improve continuation summarized in Table 2 showed promise in pivoting continuation rates in the project and similar observations have been replicated in studies in SSA [55] although this area warrants comprehensive investigation.

### 4.1 Limitations

First, using aggregate data to populate the cascade does not facilitate in‐depth longitudinal analysis of individual‐level characteristics, which might contribute to a much deeper understanding of the missed opportunities for PrEP, yet this is highly relevant to the rollout of PrEP in SSA. Second, the cascade begins with HIV testing and omits useful information on the proportion of individuals from the target population reached with PrEP information and those referred for PrEP services due to lack of reliable community data. Expanded cascades including these data points can estimate coverage rates of the target populations and identify gaps in demand creation. Third, the current analysis only presents Month‐1 and Month‐3 continuation rates. Continuation rates beyond these periods are of utmost relevance in order to document optimal PrEP use among FSW, MSM and AGYW. Finally, this paper presents a PrEP‐specific cascade although PrEP is delivered in combination with other HIV prevention interventions. Despite these limitations, this study presents worthwhile evidence about how cascades can be constructed using routinely collected and easily available data from national health systems.

## 5 CONCLUSIONS

The PrEP cascade has provided a useful framework to guide planning, implementation and monitoring performance of the Jilinde project. Jilinde has used the cascade to identify missed opportunities in implementation and course corrected to increase effective delivery of PrEP services. The Jilinde PrEP cascade provides field experiences in using a cascade framework for programme monitoring and decision‐making. By generating regular cascades, programmes can continue to monitor implementation adaptations and employ evidence‐based interventions to respond to emerging gaps. Opportunities exist for developing cascades that incorporate the holistic package of HIV combination prevention interventions.

## COMPETING INTEREST

The authors have no conflict of interest to declare.

## AUTHORS’ CONTRIBUTIONS

DW and AM conceptualized the study. JM analysed the data. DW, AM, JM, PO, GM, MK, TM and HG wrote the initial draft, MP, JR and IM contributed and revised the draft. All authors read and approved this draft.
